# Performance and Effectiveness of Diabetic Retinopathy Screening in Portugal: An Outcome-Based Evaluation

**DOI:** 10.3390/jcm14103344

**Published:** 2025-05-12

**Authors:** Inês Coelho-Costa, Amanda Silva-Pereira, Pedro Mota-Moreira, Pedro Marques-Couto, Rita Teixeira-Martins, Carolina Maia, Manuel Falcão, Rita Laiginhas

**Affiliations:** 1 Ophthalmology Department, Local Health Unit of São João, 4099-450 Porto, Portugal; ines.coelho.costa@ulssjoao.min-saude.pt (I.C.-C.); pedro.jose.moreira@ulssjoao.min-saude.pt (P.M.-M.); pedro.marques.couto@ulssjoao.min-saude.pt (P.M.-C.); rita.teixeira.martins@ulssjoao.min-saude.pt (R.T.-M.); carolina.maia@ulssjoao.min-saude.pt (C.M.); m.falcao@ulssjoao.min-saude.pt (M.F.); 2 Faculty of Medicine, University of Porto, 4200-319 Porto, Portugal; up201806049@edu.med.up.pt; 3 RISE-Health, Surgery and Physiology Department, Faculty of Medicine, University of Porto, 4200-450 Porto, Portugal

**Keywords:** diabetic retinopathy, diagnostic screening programs, predictive value of tests

## Abstract

**Background/Objectives**: Diabetic retinopathy (DR) is the leading cause of preventable blindness among working-age adults. Early detection through screening programs is essential for managing the condition and preventing visual impairment. In Portugal, the national DR screening program (DR SP) targets diabetic patients, aiming to detect DR at an early stage and refer patients requiring intervention for an ophthalmology appointment. This study aims to assess the effectiveness of the Portuguese DR SP by analyzing patients referred for a hospital appointment following a positive screening result. **Methods**: An observational retrospective cohort study was conducted at Unidade Local de Saúde de São João (ULS-SJ), including patients referred to a DR SP hospital appointment between January 2020 and December 2023. Data were collected from hospital records upon approval by the Hospital Ethics Committee. Screening and hospital diagnoses were compared for agreement. The Chi-Square test and Cohen’s Kappa were used to assess the association between screening and hospital diagnoses. **Results**: A total of 1126 patients (2251 retinographies) were analyzed. The median time from screening to hospital consultation was 63 days (Interquartile Range = 39–99), though referral times varied widely within the same classifications (ranging from 8 to 354 days). The most common screening classifications were R2 (pre-proliferative DR, 47.8%) and M1 (maculopathy, 24.6%). In eyes with DR, agreement between screening and hospital diagnoses was highest for R2 (40.1%) and M1 (32.3%), while proliferative DR (R3) showed 30% agreement. The positive predictive value (PPV) of the screening program was 55.9%, with a false positive rate of 44.1%. A statistically significant association between screening and hospital diagnoses was observed (*p* < 0.001, Chi-Square test), though Cohen’s Kappa values (0.167 Right Eye, 0.157 Left eye) indicated only slight agreement. **Conclusions**: Our study found that DR SP effectively identifies patients needing ophthalmologic evaluation with moderate diagnostic agreement and a relatively high false positive rate, leading to unnecessary referrals. While this ensures that sight-threatening cases are not missed, improvements in grader training, classification protocols, and Optical Coherence Tomography (OCT) integration could improve results. Strengthening screening adherence and optimizing referral pathways would further improve the program’s impact on early DR detection and management.

## 1. Introduction

Diabetes mellitus (DM) is one of the fastest-growing public health challenges of the 21st century. The most recent data estimates that in 2021, 537 million people worldwide had diabetes, with projections suggesting this number will rise to 643 million by 2030 and 783 million by 2045 [1]. In Europe, 1 in 11 adults have diabetes, and Portugal is among the top five European countries with the highest age-adjusted prevalence of people with DM (9.1%) [1].

Diabetic retinopathy (DR) is the most common microvascular complication of DM [2], affecting 22.27% diabetic patients worldwide (103.12 million adults globally) [3]. Studies have shown that 95% of patients with type 1 DM and 60% of those with type 2 DM, will develop DR with 20 years of disease progression [4]. It is one of the leading causes of preventable blindness and visual impairment, particularly among working-age adults, where it significantly impacts quality of life, productivity, and healthcare systems [5,6].

Morphological changes that lead to vision loss in DR are often asymptomatic and go unnoticed by patients [5,7,8]. For this reason, systematic DR screening program (DR SP) is essential for early disease detection, progression monitoring, and identifying patients who require referrals to an ophthalmologist for close follow-up and possible treatment [5,8]. DR screening was first successfully introduced in Iceland in 1980, proving cost-effectiveness [9]. As a result, various screening programs, hospital-based, regional, and national have been implemented worldwide in countries like the United Kingdom (UK), Finland, Sweden, Ireland, Denmark, and Portugal [10,11]. The choice of diagnostic methods, including direct or indirect ophthalmoscopy and fundus photography, varies according to each country’s healthcare system, with different healthcare professionals involved in the screening process [11].

In Portugal, DR screening is a national program, ideally targeting diabetic patients without DR or those with only mild, non-proliferative, DR. Screening depends on diabetes type: patients with type 1 DM start screening five years after diagnosis, whilst those with type 2 DM start screening one year after diagnosis. Following the initial screening, annual assessments are conducted [12].

Screening is performed by ophthalmic technicians at primary care facilities and consists of obtaining two retinographies per eye: one centered on the macula and the other on the optic disk. Retinographies are obtained using a non-mydriatic camera under mesopic conditions [12,13,14]. This method has been proven to have good sensibility and specificity [14]. Retinographies are uploaded to an online platform, SiiMA rastreios, which is also used for other SPs in Portugal. The images are then sent to the appropriate reading center where an ophthalmologist evaluates and classifies them, determining whether the patient should continue routine screening or requires an ophthalmology appointment at their local hospital [12,13,14]. The urgency of the referral is based on the severity of findings according to the DR screening guidelines by the Portuguese Directorate-General of Health (Direção Geral de Saúde [DGS]) [12], as detailed in Table 1, which also outlines the classifications that can be assigned during screening. At the clinic, the ophthalmologist has access to the original screening images, which is useful for contextualizing any discrepancies and for considering potential changes in the fundus that may have occurred during the interval between screening and hospital evaluation.

According to the 2023 report by the Portuguese Diabetes Society (SPD) [15], the number of individuals with diabetes covered by the DR SP in Portugal grew significantly, reaching a quarter of the diabetic population in 2018 and 2019. However, these numbers were heavily impacted by the COVID-19 pandemic, with screenings declining by over 50% in 2020. Although screening activity recovered in 2021, it remained below pre-pandemic levels. The northern Region of Portugal has the highest number of diabetic patients participating in the SP, with a total of 124,231 screenings in 2019, 67,255 in 2020, and 126,213 in 2021 [15].

This study provides a comprehensive, outcome-based evaluation of the Portuguese National Diabetic Retinopathy Screening Program (DR SP), emphasizing its relevance and public health impact. Despite the existence of a national DR screening framework, there is a notable lack of studies assessing its real-world performance and effectiveness. By retrospectively analyzing outcomes of patients referred to the DR SP consultation at the Unidade Local de Saúde de São João (ULS-SJ), a tertiary hospital in northern Portugal, following a positive screening result between 2020 and 2023, this study aims to identify both the strengths and limitations of the current program. In doing so, it contributes new evidence about program effectiveness and proposes practical, data-driven improvements to enhance care pathways for diabetic patients.

**Table 1:** Retinography classifications and recommended screening/referral intervals. Classification names follow the DR screening guidelines by Direção Geral de Saúde [12], while descriptions are adapted from the National Health Service (NHS) Diabetic Eye Screening Program grading definitions [16].

Staging of Diabetic Retinopathy (DR)	Referral and Intervention Time
**R0—No apparent DR** No DR	Repeat screening after one year
**R1—Mild non-proliferative DR (NPDR)** Microaneurysms, retinal hemorrhages +/– any exudates excluding the definition of maculopathy	Repeat screening after one year
**R2—Pre-Proliferative (moderate or severe)** “Beading” veins, venous loops, and duplications, profound multiple intraretinal microvascular abnormalities, cotton wool spots	Ophthalmology consultation within 2 months
**R3—Proliferative DR** Disk neovascularization, retinal neovascularization, vitreous or pre-retinal hemorrhage, pre-retinal fibrosis +/− traditional retinal detachment	Ophthalmology consultation within <1 month
**M1—Maculopathy** Exudates within one disk diameter (DD) of the foveal center, circinate or grouped exudates in the macular area, retinal thickening within 1 DD of the foveal center, any microaneurysm or hemorrhage within 1 DD of the foveal center.	Ophthalmology consultation within <1 month
**V1—High-risk proliferative DR (V1 PDR), vitreous hemorrhage, or tractional retinal detachment**	Ophthalmology consultation within <15 days
**ICN—Inconclusive or comorbidities**	Ophthalmology consultation
**Treatment Monitoring**	
**P0—Stable Panretinal Photocoagulation**	Repeat at the end of 1 year
**P1—Insufficient Panretinal Photocoagulation**	Requires additional treatment

## 2. Methods

An observational retrospective cohort study was conducted, including all patients referred for a DR SP hospital appointment at ULS-SJ following a positive screening result between 7 January 2020 and 5 December 2023. A list of all patients who attended a hospital appointment during this period was obtained with the assistance of the hospital’s informatics department, which used codes RRD 34447 and RRD 34462 to identify appointments related to DR screenings. The inclusion criterion was attendance at a hospital appointment during the study period following a positive DR screening. The exclusion criteria were incomplete medical records and appointments misclassified as DR screening consultations but were actually unrelated (e.g., general ophthalmology or follow-up for other conditions).

Hospital records were analyzed to collect demographic and clinical data. The retrieved information included patient sex, age, diabetes type, insulin treatment status, duration of diabetes since diagnosis, date of screening, screening results for both the right and left eyes, total number of screening visits over the years, DR classification for each eye at the hospital appointment, and any additional ophthalmic diagnoses made during the ophthalmology consultation.

IBM^®^ SPSS^®^ Statistics version 27 software (IBM Corp., Armonk, New York, NY, USA) was used for statistical analysis. Quantitative variables were summarized using means and standard deviations (SD) for normally distributed data and medians with interquartile ranges (IQR) for non-normally distributed data. Normality was assessed using the Kolmogorov–Smirnov test. Categorical variables were described as frequencies and percentages. The time interval between screening and hospital appointments was calculated in days and analyzed across different worst eye screening classifications. Cross-tabulations were performed to evaluate the agreement between screening classifications and hospital diagnoses. Cohen’s Kappa statistics were used to assess the consistency of classification, while the Chi-Square test was applied to determine the statistical association between the two. Statistical significance was set at *p* < 0.05.

This study was conducted following approval from the Hospital Ethics Committee of ULS-SJ. The committee reviewed and approved the study protocol (No. 76/2024), ensuring that it adhered to ethical standards and complied with applicable guidelines for research involving human subjects. As this was a retrospective analysis of existing clinical records, the requirement for informed consent was **waived** by the Ethics Committee. All data were anonymized and handled in accordance with data protection regulations to ensure patient confidentiality.

## 3. Results

### 3.1. Sample

During the study period, a total of 1277 DR SP consultations were conducted at ULS-SJ. One hundred fifty-one patients were excluded due to incomplete medical records or having incorrectly coded appointments. This resulted in a final sample of 1126 patients which included 2251 retinographies (notably, one patient was monocular). Among the patients studied, 58.8% (n = 662) were male and the mean sample age was 66.6 years (SD = 11.33). A significant majority, 93.4% (n = 1052), had type 2 DM and 3.4% (n = 38) were diagnosed with type 1 DM. Other types of diabetes, including maturity-onset diabetes of the young (MODY), type 1B, and autoimmune diabetes, accounted for 0.4% (n = 5) of the cohort. The mean time since diabetes diagnosis, up until screening date, was 16.07 years (SD = 9.50), with no information regarding disease duration available for 7.2% (n = 81) of patients. 44.6% (n = 327) of patients were treated with insulin and 55.4% (n = 406) were treated exclusively with oral antidiabetics.

### 3.2. Diabetic Retinopathy Screening

**Figure 1:**
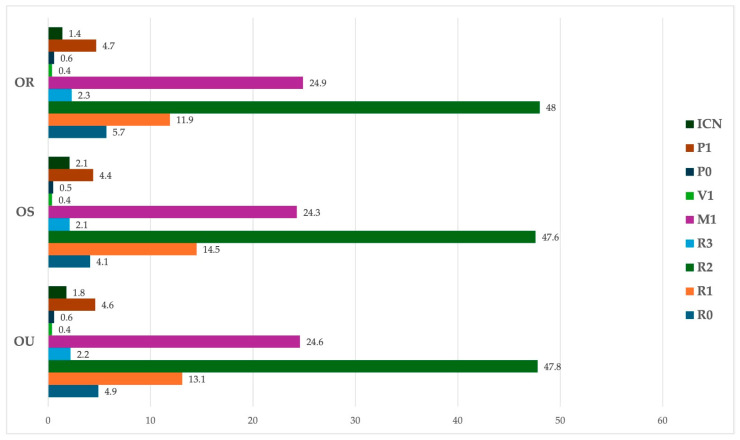
Screening results for the right (OR), left (OS), and both eyes (OU). Values in %. R0-no apparent DR; R1-mild non-proliferative DR; R2-pre-proliferative DR; R3-proliferative DR; M1-maculopathy, V1-high-risk proliferative DR, vitreous hemorrhage or tractional retinal detachment; P0-stable panretinal photocoagulation; P1-insufficient panretinal photocoagulation; ICN-inconclusive.

Screening results are shown in Figure 1. The largest proportion of retinographies were classified as R2 (pre-proliferative DR [47.8%, n = 1077]), followed by M1 (maculopathy [24.7%, n = 555]) and R1 (mild non-proliferative DR (NPDR) [13.1%, n = 296]). Less than 1% (n = 10) of analyzed retinographies were classified as V1 (high-risk proliferative DR/vitreous hemorrhage/tractional retinal detachment) or R3 (proliferative DR), the most severe classifications.

The median time between a positive screening and the ophthalmology appointment for the whole sample was 63 days (IQR = 39–99). Referral times stratified by worst eye retinography classification are presented in Table 2. For patients classified as R2 (pre-proliferative diabetic retinopathy), which was the most frequent referral category, the median referral time was 66 days. However, a wide range of referral times was observed within this classification, with waiting times spanning from 8 to 354 days. Similar variability in referral times was observed across other classifications (Table 2).

On average, patients included in our sample underwent 3.5 screenings (SD = 1.70) since their diabetes diagnosis, corresponding to approximately one screening every four years.

### 3.3. Results of the Diabetic Retinopathy Consultation

**Figure 2:**
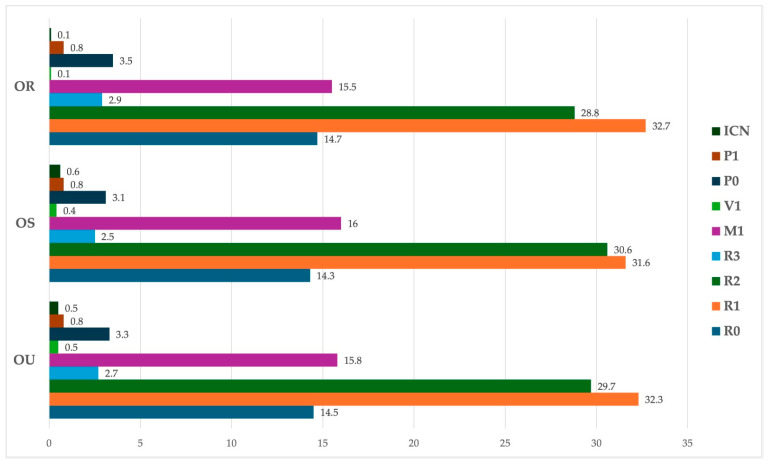
Diabetic Retinopathy hospital ophthalmology appointment results for the right, left and both eyes. Values in %. DME-diabetic macular edema requiring treatment.

With regard to DR staging at the hospital appointment following a positive screening result (Figure 2), the most common diagnosis was mild non-proliferative DR (32.2%; n = 724), followed by pre-proliferative DR (29.7%; n = 668). Also, a significant number of eyes were diagnosed with maculopathy or diabetic macular edema (DME) requiring treatment (15.8%; n = 356), with other classifications representing smaller proportions.

During the hospital appointment, apart from DR, 17.3% (n = 389) of eyes were diagnosed with another ophthalmic condition. The most common diagnoses were cataract (5.3%; n = 119) and epiretinal membrane (3.4%; n = 77). Other diagnoses included age related macular degeneration (1.4%; n = 32) and venous occlusion (1.1%; n = 25). Less frequent diagnoses, such as retinoschisis and nevus, accounted for less than 1% of cases.

### 3.4. Screening Program Analysis

When analyzing the agreement between screening classifications and hospital diagnoses, in eyes that warranted referral, the highest agreement was observed for R2, with 40.1% (n = 432) of eyes maintaining the same classification at the hospital visit. Maculopathy (including diabetic macular edema requiring treatment) had an agreement of 32.3% (n = 179), while proliferative DR showed an agreement of 30% (n = 15). For V1 (high-risk proliferative DR), the diagnosis was concordant in 10% (n = 1) of eyes and for P1 (insufficient photocoagulation) the accuracy was 11.7% (n = 12).

With regard to the remaining patients that were screened as R2 and did not match the hospital diagnosis, 34.1% (n = 367) were downgraded to mild NPDR, 13.2% (n = 142) were diagnosed with maculopathy (including DME requiring treatment), 8.5% (n = 92) were found to have no DR, and smaller proportions were classified as proliferative DR (2.7%; n = 29) or high-risk PDR (0.2%; n = 2).

Eyes screened as R0 and R1 showed the highest agreement (58.3% [n = 63] and 52.7% [n = 156], respectively), although these classifications did not require referral. In almost all cases, their presence in the hospital dataset was due to these patients having been referred due to a worse classification in the fellow eye, rather than the R0 or R1 diagnosis itself. In fact, only four patients were wrongly referred for a hospital appointment by not meeting referral criteria (one patient with R0 classification for both eyes and four patients with the worst-eye classification being R1).

Since the study sample consisted only of patients who screened positive, sensitivity and specificity could not be determined. The positive predictive value (PPV) of the screening program was 55.9%, with a false positive (FP) rate of 44.1%. Of the 1126 patients referred for a hospital ophthalmology appointment, 629 were confirmed to have DR requiring treatment or warranting close ophthalmologic follow-up, defined as a hospital diagnosis of pre-proliferative DR or worse.

A Chi-Square test demonstrated a statistically significant association between screening and hospital diagnoses for both eyes (*p* < 0.001). However, Cohen’s Kappa values of 0.167 for the right eye and 0.157 for the left eye indicate only a slight agreement between the two methods.

## 4. Discussion

The purpose of the present study was to assess the performance and effectiveness of the Portuguese DR SP by analyzing patients referred to ULS-SJ following a positive screening result.

By including all patients referred for a hospital ophthalmology appointment during the study period, we were able to obtain a robust dataset (1126 patients and 2251 retinographies) and ensure a longitudinal representation by including four years of data. Most patients analyzed were elderly and had type 2 diabetes, with an average disease duration of approximately 16 years, which is in line with previous studies [13,14]. Additionally, the samples’ gender distribution was balanced (58.9% male and 41.1% female), ensuring a low risk of gender-based biases in screening and referral.

Substantial variability was found regarding the time from screening to the hospital appointment, regardless of the screening diagnosis. Although referral times varied across DR classifications, for the most common classification, R2, they were only slightly superior to the goals outlined by the DGS (Table 1). Nonetheless, data from the Northen Portugal regional health administration (ARS Norte [2023]), shows that the median waiting time for an ophthalmology consultation at ULS-SJ, when referred by a general practitioner (GP), is 3.2 months [17]. This suggests that SP is effective in the early referral of patients with DR compared to standard GP referrals. However, delays remain, warranting optimization in referral prioritization.

Patients in the sample underwent screening approximately once every four years instead of annually, pointing to limited adherence to the program’s recommended yearly screening. Additionally, according to the SPD [15], coverage of the DR SP is only around 40%, meaning a large proportion of diabetic patients do not participate in regular screenings. Given that DR is often asymptomatic in early stages, increasing screening adherence is essential. Greater efforts should be made to educate both patients and healthcare professionals on the importance of routine DR screening, emphasizing its role in preventing vision-threatening complications [5,8].

Our results showed that the agreement between screening classifications and hospital diagnoses varied considerably across different DR stages. The highest agreement was observed for R0 and R1 (58.3% and 52.7%, respectively), which do not require referral, suggesting that the screening program is fairly reliable in identifying patients without significant disease. However, for R2 (pre-proliferative DR), agreement was lower, with 59.9% of cases being reclassified at the hospital visit. Of these, the majority were downgraded to mild NPDR (34.1%), suggesting that a significant proportion of R2 cases may have been overestimated in screening. A total of 13.2% of R2 screenings were reclassified as maculopathy (including DME requiring treatment), highlighting the overlap between different DR stages and the difficulty in distinguishing early maculopathy from pre-proliferative DR using retinography alone. For more severe DR stages, agreement was variable. Proliferative DR (30%) and maculopathy (32.3%) showed moderate agreement, while high-risk PDR (10%) and insufficient photocoagulation (11.7%) had low agreement, likely due to the inherent limitations of retinography in detecting neovascularization, vitreous hemorrhage, or laser treatment adequacy. The low agreement observed for maculopathy (32.3%) is particularly concerning, as this classification includes DME requiring treatment. The misclassification of maculopathy may stem from the difficulty in detecting subtle retinal thickening or differentiating early macular changes from true DME, reinforcing the need for additional imaging techniques to improve accuracy in cases where retinography alone may be insufficient.

The tendency to overestimate DR severity, particularly in R2 cases, contributed to the FP rate of 44.1%, with nearly half of referred patients not requiring intervention or close follow-up. While this may lead to unnecessary referrals, it ensures that patients with sight-threatening disease are not overlooked, which is preferable to the risk of underdiagnosis and delayed treatment. Despite these challenges, a statistically significant association between screening results and hospital diagnoses was observed (*p* < 0.001, Chi-Square test) confirming the program’s role in identifying patients needing further evaluation. However, Cohen’s Kappa values (0.167 OR, 0.157 OS) indicated only slight agreement, suggesting that while the screening correctly identifies patients needing evaluation, its ability to precisely classify DR severity is limited. This discrepancy may be influenced by image quality variability, grader experience, or differences in clinical assessment methods, emphasizing the need for enhanced grader training and standardized classification protocols. Although retinographies are obtained using a non-mydriatic camera under mesopic conditions, several factors—such as small pupil size or media opacities—can interfere with image clarity and impact diagnostic accuracy. The integration of additional diagnostic tools, in particular Optical Coherence Tomography (OCT), into the screening program could significantly reduce misclassification rates, especially for maculopathy cases, where OCT is essential for detecting retinal thickening and differentiating DME from other macular changes. The use of OCT could improve the accuracy of DR severity classification, minimize unnecessary referrals, and ultimately enhance the overall effectiveness of the screening program.

Beyond DR, the DR SP also facilitated the identification of other ocular conditions, reinforcing its broader value in eye health monitoring. In this study, 17.3% of eyes had additional diagnoses, with cataracts and epiretinal membrane being the most frequent additional findings. Other pathologies, including age-related macular degeneration and venous occlusion, were also detected, some of which could have gone undiagnosed without screening. While the program primarily targets DR detection, these findings highlight its role in identifying other conditions that also require timely intervention; therefore, strengthening screening protocols for the referral of cases where non-DR pathologies are identified could enhance the program’s impact on overall eye health.

As previously mentioned, DR screening has been implemented worldwide since it has proven to be cost-effective in the early identification of DR [9]. The UK’s National Health Service (NHS) Diabetic Eye Screening Program is widely recognized as one of the largest SPs in Europe [11], with a reported 83% coverage rate in 2018/2019 [18], with all diabetic individuals aged 12 years and over being invited for an annual DR screening appointment [18,19]. In 2023, a biennial screening model for low-risk patients was adopted to optimize healthcare resources, an approach supported by cost-effectiveness studies [20,21,22]. Given the low adherence to annual screening observed in our cohort, a similar shift to biennial screening for low-risk patients in Portugal could free up resources.

In the NHS Diabetic Eye SP, screeners are required to undertake a diploma in retinal screening, which trains them in acquiring high-quality images, performing visual acuity tests, and safely administering dilation drops. Additionally, each image is independently evaluated for adequacy and accessibility, ensuring accurate interpretation. The program also enforces strict quality assurance, including key performance indicators, external audits, and mandatory monthly grader assessments, with additional training required for underperforming graders. If a provider fails to meet standards, the service may be reassigned [18,19]. Adopting similar training strategies and quality control measures in Portugal, such as regular grader evaluations, standardized classification protocols, and retinography quality assurance, could reduce misclassification rates and enhance screening accuracy, ensuring a more reliable and effective program.

In addition, other countries, such as Denmark and Israel, have also established successful DR screening programs with high coverage rates. According to the World Health Organization [18], in Denmark, 99% of patients with diabetes had undergone at least one DR screening within the previous five years (2018–2019), while in Israel, 72.5% of patients diagnosed with diabetes underwent eye screening in 2018. Sweden has also implemented a structured and effective DR SP, regionally organized but guided by national standards and integrated with the Swedish National Diabetes Register (NDR). Screening is conducted using fundus photography, and follow-up intervals are risk-based. High coverage and centralized data collection have made the Swedish model a strong example of how digital infrastructure can support equitable and efficient DR screening across a decentralized system [11], offering a potential model for improving coordination and long-term monitoring. On the other hand, countries like Australia operate a predominantly opportunistic screening model that is less centralized, often resulting in inconsistent access. Several barriers, including limited access to retinal imaging equipment, time constraints in general practice, insufficient training, and unclear referral pathways, hinder widespread implementation. These factors contribute to variability in screening uptake and continuity of care, particularly in underserved communities [23]. While Portugal benefits from a nationally coordinated program, these challenges highlight the importance of reinforcing organization and training to ensure more consistent delivery across all regions.

Despite room for improvement, the Portuguese SP demonstrates several notable strengths when compared to other countries. First, it is incorporated into the National Health Service, ensuring universal and equitable access without the financial barriers often seen in countries reliant on private insurance. Second, the integration of DR screening into primary care facilities facilitates patient follow-up and referrals, reducing the risk of dropouts or missed diagnoses. Third, remote image acquisition and centralized grading by ophthalmologists enable broad geographic coverage, including rural areas. This strategy has supported the efficient use of limited resources.

Despite having a robust study, we identified some limitations. By only including patients from one tertiary center, our findings may not fully reflect the national reality of DR screening, as there could be geographic and epidemiological differences across regions. Variations in DR prevalence, healthcare access between urban and rural areas, and differences in screening implementation may affect the generalizability of our results to other parts of the country. In addition, our sample did not account for those patients who screened as negative and thus were not referred. Additionally, our study analyzed data from 2020 to 2023, which, while providing the most recent insights into the screening program also includes the COVID-19 pandemic period (2020–2022). Given that healthcare services were significantly disrupted during this time, with delays in screenings and consultations, the pandemic may have had some impact on our findings.

## 5. Conclusions

Despite the above mentioned challenges, the DR SP successfully facilitated the early referral of 55.9% of patients with DR requiring treatment or close monitoring, expedited access to ophthalmologic consultations, and enabled the detection of other ocular diseases that might have otherwise been delayed in diagnosis and treatment. Strengthening grader training and implementing standardized quality control measures could reduce misclassification rates and improve screening reliability. Addressing low screening adherence—through patient education initiatives and improved coordination with primary care providers should also be a priority. Additionally, exploring a biennial screening model for low-risk patients could allow better resource allocation toward high-risk patients, optimizing the DR SP and its role as a crucial tool in the early detection and long-term impact on diabetic eye care.

## Figures and Tables

**Table 2:** Time in days from the screening to the hospital appointment, according to worst eye retinography classification.

Worst Eye Retinography Classification	Number of Patients	Median (IQR), Min.–Max.
**R0**	1	301
**R1**	4	38.5 (77), 23–121
**R2**	605	66 (57), 8–354
**R3**	35	74 (77), 11–170
**M1**	411	54 (65), 10–323
**V1**	8	68.5 (59), 44–120
**P1**	62	79 (58), 12–262

## Data Availability

The data are not publicly available but can be obtained from the corresponding author upon reasonable request and with institutional approval.

## Abbreviations

The following abbreviations are used in this manuscript:

ARS	Regional Health Administration
COVID-19	Coronavirus Disease 2019
DGS	Direção Geral de Saúde
DM	Diabetes Mellitus
DME	Diabetic Macular Edema
DR	Diabetic Retinopathy
GP	General Practitioner
IQR	Interquartile Range
MODY	Maturity-Onset Diabetes of the Young
NHS	National Health Service
NPDR	Non-proliferative Diabetic Retinopathy
OCT	Optical Coherence Tomography
OR	Right Eye
OS	Left Eye
OU	Both Eyes
PDR	Proliferative Diabetic Retinopathy
PPV	Positive Predictive Value
SD	Standard Deviation
SP	Screening Program
SPD	Portuguese Diabetes Society
UK	United Kingdom
ULS-SJ	Unidade Local de Saúde de São João
